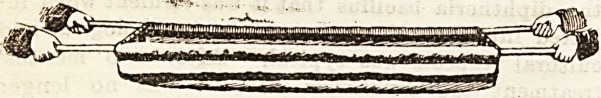# New Appliances and Things Medical

**Published:** 1896-05-23

**Authors:** 


### NEW APPLIANCES AND THINGS MEDICAL.

### FOLDING CRADLE STRETCHER.

It may be remembered that some time ago we gave a description of a lifting seat and carrying chair, the invention of an invalid lady (see The Hospital for November 30th, 1895, p. 155), which combined many excellencies for its special purpose. We now give a sketch of a folding stretcher, the outcome of the same lady's inventive brain, which is intended to take a patient in a recumbent position, and by its shape to prevent any possibility of slipping or falling out, or rather to obviate the fear of such a catastrophe on the part of the occupant. The stretcher is made of strong canvas, and in three pieces, "one long piece, wide enough to form the stretcher and its sides, and two other pieces, one for the head and one for the foot . . . . These two pieces govern the width of the stretcher and the height of the sides, being made as wide as the stretcher is to be, and as deep as the sides." The ends have stiff rods to keep the proper width, like a hammock, and fasten on or take off as required. The stretcher goes quite flat when laid on the ground. Experiments made at an ambulance class at Ipswich resulted in general satisfaction, both to carriers and carried, the latter testifying to a most comfortable feeling of security. Canvas stretchers, after much washing, *have* been known to collapse at a critical moment, and let the patient through, but to prevent such a catastrophe it only needs to exercise reasonable care in renewing the canvas. The makers are Fred. Fish and Son, Suffolk House, Ipswich. The price of the stretcher, with poles, as seen in the accompanying drawing, is 13s. 6d. Messrs. Fish offer special terms to hospitals.

## Figures and Tables

**Figure f1:**